# Complete Chloroplast Genome Sequence of Tartary Buckwheat (*Fagopyrum tataricum*) and Comparative Analysis with Common Buckwheat (*F*. *esculentum*)

**DOI:** 10.1371/journal.pone.0125332

**Published:** 2015-05-12

**Authors:** Kwang-Soo Cho, Bong-Kyoung Yun, Young-Ho Yoon, Su-Young Hong, Manjulatha Mekapogu, Kyung-Hee Kim, Tae-Jin Yang

**Affiliations:** 1 Highland Agriculture Research Institute, National Institute of Crop Science, Rural Development Administration, Pyeongchang, South Korea; 2 Department of Plant Science, College of Agriculture and Life Sciences, Seoul National University, Seoul, South Korea; 3 Phygen Genomics Institute, Gwanak Century Tower, Kwanak-gu, Seoul, South Korea; Austrian Federal Research Centre for Forests BFW, AUSTRIA

## Abstract

We report the chloroplast (cp) genome sequence of tartary buckwheat (*Fagopyrum tataricum*) obtained by next-generation sequencing technology and compared this with the previously reported common buckwheat (*F*. *esculentum* ssp. *ancestrale*) cp genome. The cp genome of *F*. *tataricum* has a total sequence length of 159,272 bp, which is 327 bp shorter than the common buckwheat cp genome. The cp gene content, order, and orientation are similar to those of common buckwheat, but with some structural variation at tandem and palindromic repeat frequencies and junction areas. A total of seven InDels (around 100 bp) were found within the intergenic sequences and the *ycf1* gene. Copy number variation of the 21-bp tandem repeat varied in *F*. *tataricum* (four repeats) and *F*. *esculentum* (one repeat), and the InDel of the *ycf1* gene was 63 bp long. Nucleotide and amino acid have highly conserved coding sequence with about 98% homology and four genes—*rpoC2*, *ycf3*, *accD*, and *clpP*—have high synonymous (Ks) value. PCR based InDel markers were applied to diverse genetic resources of *F*. *tataricum* and *F*. *esculentum*, and the amplicon size was identical to that expected *in silico*. Therefore, these InDel markers are informative biomarkers to practically distinguish raw or processed buckwheat products derived from *F*. *tataricum *and *F*. *esculentum*.

## Introduction

Chloroplasts are essential organelles in plant cells that perform photosynthesis, in addition to other functions including synthesizing sugars, pigments, and certain amino acids. The chloroplast (cp) is considered to have originated from an ancestral endosymbiotic cyanobacteria. In addition to the larger dominant genome located in the nucleus of plant cell, chloroplasts contain their own independent genome encoding a specific set of proteins. The non-recombinant nature of the cp genome makes it a potentially useful tool in genomics and evolutionary studies. Although the cp genome is highly conserved in vascular plants, evolutionary hotspots such as single nucleotide polymorphisms [SNPs] and insertion/deletions [In/Dels] resulting from inversions, translocations, rearrangements and copy number variation of tandem repeats have been found in many plants [1]. As such, these SNPs and In/Dels are useful as molecular markers as the cp genome is highly conserved within the species. Further, cp DNA can be easily extracted from samples because of the high copy number. The small size of the cp genome makes it suitable for complete sequencing and the data can be further applied to phylogeny construction [2], DNA bar coding [3], and transplastomic studies [4]. Complete cp DNA sequencing began in 1991 [5] and to date cp genomes of various algae and plants, including crop species, have been reported (CpBase: http://chloroplasr.ocean.washington.edu).

Until recently, cp genome sequencing was a costly and time-consuming process. The majority of such research, therefore, has been limited to sequencing a small portion of the cp genome, which in many cases is insufficient for determining evolutionary relationships, thereby limiting its utility for plant evolutionary and genomic studies. As complete cp genome sequences harbor sufficient information, sequencing of whole cp genomes is essential for the comparison and analyses of diversifications among plant species. The advent of next-generation sequencing (NGS) has made it considerably cheaper and easier to sequence complete cp genomes. NGS is advantageous as it provides extremely high yield and the opportunity for multiplexing when investigating whole-cp genomes, rather than targeting individual regions [6,7]. NGS allows potentially hundreds of flowering plant cp genomes to be sequenced simultaneously, significantly reducing the per-sample cost of cp genome sequencing [8].

Buckwheat (*Fagopyrum* species) belonging to Polygonaceae, a member of knotgrass is an annual herbaceous plant. Buckwheat is classified into twenty species, is largely centered in the Eurasian region, and is mainly grown in the highlands [9,10]. It is divided into two groups—cymosum and urophyllum, based on the morphology and cp genome [11]. The cymosum group comprises *F*. *esculentum*, *F*. *tataricum*, *F*. *cymosum*, *and F*. *homotropicum*, which are characterized according to the flowering calyx (persistent perianth) and achene. The urophyllum group comprises *F*. *urophyllum*, which is characterized by a glossy calyx. Among these, common buckwheat (*F*. *esculentum*) and tartary buckwheat (also known as bitter buckwheat (*F*. *tataricum*), are used in various dietary preparations and are mainly grown in South Korea, Japan, and China [12]. Because of the nutritional value of tartary buckwheat, the cultivated area in South Korea has increased in recent years [13]. Bitter buckwheat is a particularly rich source of rutin compared to common buckwheat, which helps reduce intra-vascular cholesterol, high blood pressure, and diabetes. Rutin is also reported to have a crucial role in pharmaceutical research [14,15,16].

The complete cp genome of *Fagopyrum* may provide useful information for phylogenetic comparisons with the related species. To date there have been few cp genome sequencing studies performed in buckwheat. The complete cp genome sequence of a wild ancestor of cultivated buckwheat *F*. *esculentum* spp. *ancestrale* was reported using amplification, sequencing, and annotation (ASAP) method [17]. Here, we present the complete cp genome sequence of tartary buckwheat (*F*. *tataricum*) by using NGS and comparative analysis with common buckwheat (*F*. *esculentum*). To the best of our knowledge, this is the first report of the complete cp genome sequence of tartary buckwheat. Comparative analysis between two *Fagopyrum* species could reveal the evolution of each species and provide practical biomarkers to authenticate common and bitter buckwheat products.

## Materials and Methods

### Plant material and DNA extraction

Genetic resources of tartary buckwheat (*F*. *tataricum*) and common buckwheat (*F*. *esculentum*) were obtained from the National Agrodiversity Center of the Rural Development of Administration (http://genebank.rda.go.kr), Korea (S1 Table). For the cp genome sequencing, ten *F*. *tataricum* cv. Daegwan 3–3 (cultivar developed by HARC) plants were raised from the seeds of a single mother plant. Total genomic DNA was isolated from approximately 100 mg of fresh leaves using the DNeasy Plant MiniKit (Qiagen, CA, USA).

### Next generation sequencing and chloroplast genome assembly

Genomic DNA was used for sequencing by an Illumina Hiseq2000 (Illumina, San Diego, CA, USA) platform in Macrogen (Macrogen, Seoul, Korea) and cp genome was obtained by *de novo* assembly of the low coverage whole genome sequence via a bioinformatics pipeline (http://phyzen.com). A 500 bp paired end library was made according to the Illumina PE standard protocol and generated 5,234,883,126 bp of total reads with a 101 bp average read length. Raw reads with Phred scores of 20 or less were removed from among the total PE reads using the CLC-quality trim tool and *de novo* assembly was conducted using trimmed reads by a CLC genome assembler (ver. 4.06 beta, CLC Inc, Rarhus, Denmark) with parameters of minimum (200 to 600 bp) autonomously controlled overlap size. The principal contigs (Ctgs) representing the cp genome were retrieved from the total Ctgs using Nucmer [18] with the cp genome sequence of common buckwheat (NC_010776) as reference sequence. The representative cp Ctgs were arranged in order based on BLASTZ analysis [19], with the reference sequence and connected into a single draft sequence by joining overlapping terminal sequences. Gene annotation was conducted using DOGMA [20] and manual editing through comparison with the reported cp genome sequence of common buckwheat (NC_010776). The circle map of the *F*. *tataricum* cp genome was obtained using OrganellarGenomeDRAW software (OGDRAW, http://ogdraw.mpimp-golm.mpg.de) [21].

### Comparative analysis with common buckwheat

The cp genome sequence (NC_010776) of common buckwheat was obtained from the National Center for Biotechnology Information (NCBI) and summary information was obtained from CpBase (http://chloroplast.ocean.washington.edu). mVISTA was used to compare similarities between two Fagopyrum species [22]. Nucleotide and amino acid diversity was analyzed by BLASTN and BLASTP. Tandem and palindrome repeats were analyzed using REPuter and einverted (http://emboss.bioinformatics.nl) with 90% similarity and minimum size (20 bp), respectively. Ks and Ka values were calculated with PAL2NAL (http://www.bork.embl.de) [23].

### PCR amplification and sequencing for the validation of InDel markers

We selected around 100 bp InDel regions based on the mVISTA similarities for PCR and the primers were designed by Primer3 (S2 Table). To amplify InDel regions, 20 ng of genomic DNA was used in a 20 μl PCR mixture containing 2X TOPsimple preMix-nTaq master mix (Enzynomics, Seoul, South Korea) consisting of 0.2 U/μl n-taq DNA polymerase, 3 mM Mg^2+^, 0.4 mM each dNTP mixture with 10 pMol of each primer. The PCR reaction was performed in a thermocycler (Veriti, Applied Biosystems, CA, USA) using the following cycling parameters: 94°C (5 min); 35 cycles of 94°C (30 s), 55°C (30 s), 72°C (1 min); and final extension at 72°C (10 min). PCR products were analyzed by 1.5% agarose gel electrophoresis and detected by DNA LoadingSTAR (DyneBio, Gyeonggi-do, South Korea). PCR products were sequenced by direct sequencing by Bionics Co. (Bionics Co., Seoul, South Korea). InDel regions from GenBank, NGS data and Sanger sequencing results were aligned using CLUSTALW.

### Ethics Statement

#### Experimentation

All materials were obtained from Agrodiversity GeneBank in South Korea (http://genebank.rda.go.kr).

#### Publication

We comply with best practices in publication ethics, specifically regarding authorship, dual publication, plagiarism, figure manipulation, and competing interests.

## Results

### Complete chloroplast genome sequence of *F*. *tataricum*

**Fig 1:**
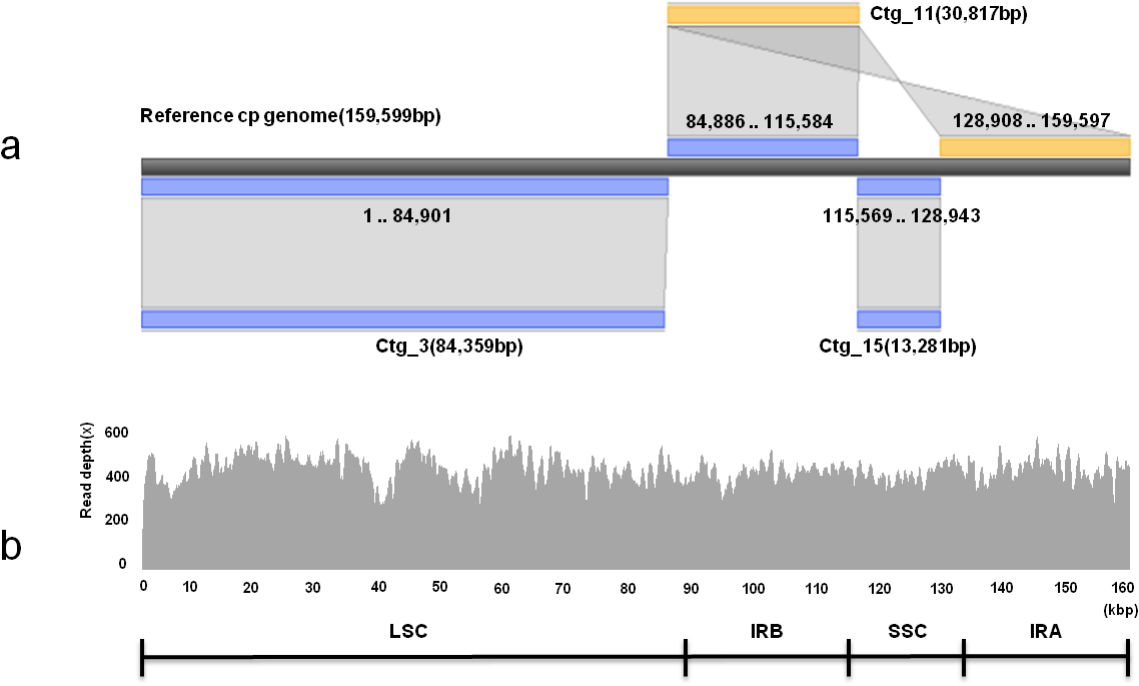
Assembly result of the complete chloroplast (cp) genome sequence of *F*. *tataricum*. a. Three representative contigs (Ctgs) for the cp genome of tartary buckwheat and comparison with the corresponding regions of the common buckwheat cp genome. **b**. Mapping of WGS raw reads onto the completed cp sequence of tartary buckwheat. The principal structure of tartary buckwheat cp genome as represented by LSC, IRa, SSC, and IRb regions. Three representative Ctgs for the cp genome, Ctgs #3, #11, and #15, were arranged in an order based on BLASTZ analysis (http://nature.snu.ac.kr/tools/blastz_v3.php) and overlapping between adjacent Ctgs. Blue and yellow bars indicate Ctgs matching the reference sequence in forward and reverse orientations, respectively, and the matching nucleotide positions are denoted at the reference cp sequence.

Sequencing of the complete cp genome of tartary buckwheat was performed by NGS technology. From the *de novo* assembly of 5,234,883,126 bp whole genome paired end sequences, we obtained three contigs covering the entire reported cp genome sequence of common buckwheat (NC_010776). Three contigs showed approximately 20-bp overlap between the flanking contigs and were joined as one single circular complete sequence by manual editing (Fig 1A). Putative assembly errors were curated by mapping of 371.40× raw reads on the final assembly (Fig 1B). Further validation was conducted by PCR and ABI sequencing of several regions that were reported to contain InDels, comparing our assembled sequence of tartary buckwheat (*F*. *tataricum*, GenBank accession no. KM201427) and the previously reported sequence of common buckwheat (*F*. *esculentum*, GenBank accession no. NC 010776). We found that NGS-based assembly of the complete cp genome were 100% identical with the ABI sequences of the selectively amplified regions.

### Comparative analysis of chloroplast genome

The tartary buckwheat complete cp genome has a total sequence length of 159,272 bp, which is 327 bp shorter than that of the common buckwheat genome (159,599 bp). The cp genome of both of these species share the common feature of containing two inverted repeats, which divide the whole genome into a large single copy region (LSC) and a small single copy region (SSC). The LSC is comprised of 84,398 bp in tartary buckwheat and 84,888 bp in common buckwheat, whereas the SSC is 13,292 bp and 13,343 bp and the inverted repeat region (IR) is 61,532 bp and 61,368 bp in tartary and common buckwheat, respectively. Tartary and common buckwheat contained CDS base total of 85,823 bp (average CDS length of 987 bp) and 82,830 bp (average CDS length of 986 bp) respectively. The total RNA bases were 11,942 (tartary) and 11,950 (common) and the overall GC-content was similar in each species (37.9% and 38% in tartary and common buckwheat respectively) with a GC skew of -0.016 (tartary) and 0.02 (common). Total repeat bases accounted for 1,056 and 804 bp, with average repeat lengths of 48 and 45 bp in tartary and common buckwheat respectively. The average intergenic distance was 495 bp and 502 bp in tartary and common buckwheat respectively (Table 1).

**Table 1:** Comparison of the complete chloroplast genome contents of *F*. *esculentum* and *F*. *tataricum*.

	*F*. *esculentum*	*F*. *tartaricum*
Total Sequence Length (bp)	159,599	159,272
Large Single Copy (bp)	84,888	84,398
Inverted Repeat Region (bp)	61,368	61,532
Small Single Copy (bp)	13,343	13,292
GC Content (%)	38.0	37.9
GC Skew	0.02	-0.016
Total CDS Bases (bp)	82,830	85,823
Average CDS Length (bp)	986	987
Total RNA Bases (bp)	11,950	11,942
Total Repeat Bases (bp)	804	1,056
Average Repeat Length (bp)	45	48
Average Intergenic Distance (bp)	502	495

**Fig 2:**
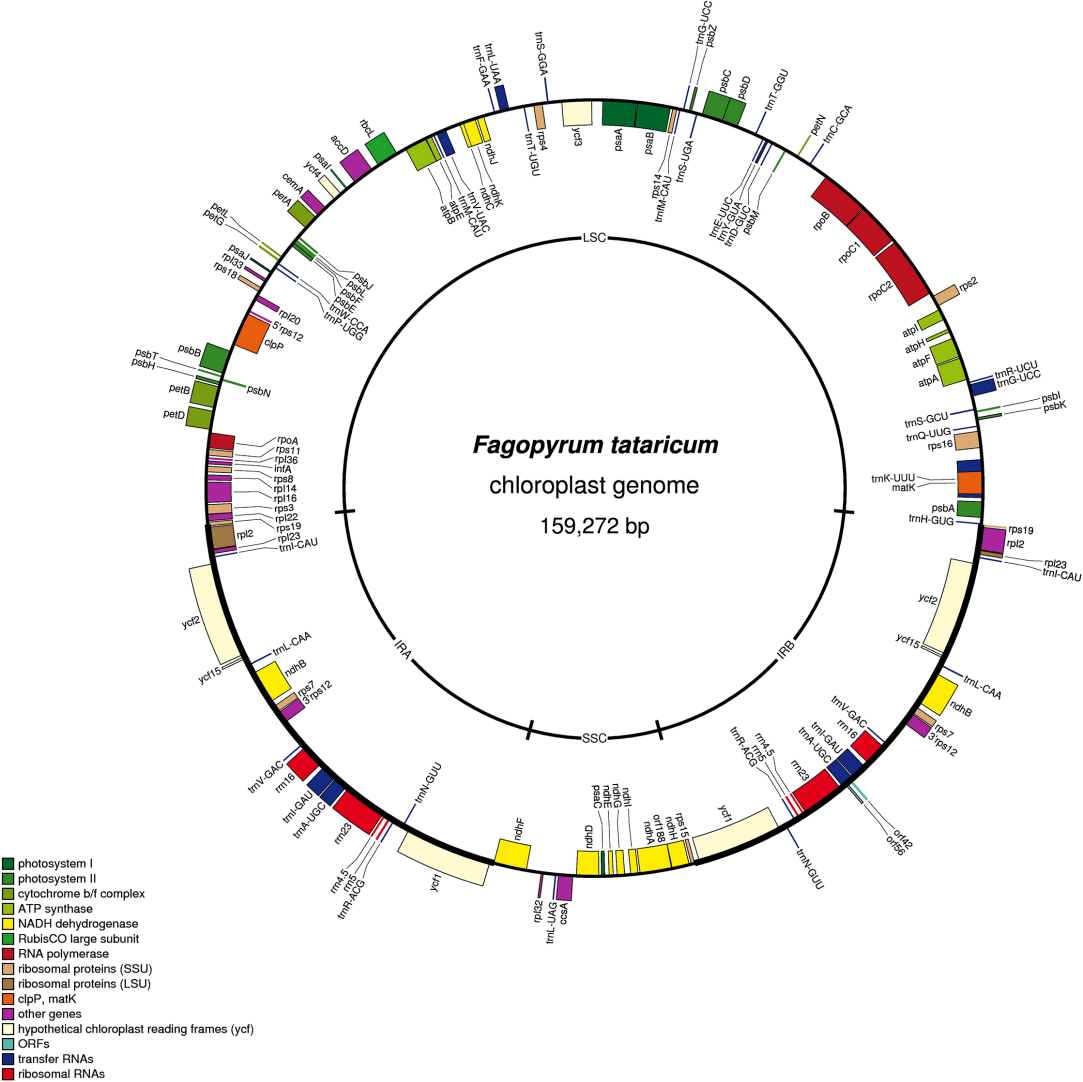
Circular gene map of the *F*. *tataricum* chloroplast (cp) genome. Genes shown inside the circle are transcribed clockwise, and those outside the circle are transcribed counterclockwise.

The gene content, order, and orientation of the *F*. *tataricum* cp genome were similar to those of common buckwheat (Fig 2). The *F*. *tataricum* cp genome has a total of 114 genes including 81 protein coding genes, 29 transfer RNA (tRNA) genes and 4 ribosomal RNA (rRNA) genes. Protein coding genes include photosynthesis related genes (the majority), in addition to transcription and translation related genes (S3 Table). The LSC region of the *F*. *tataricum* cp genome has 63 protein coding genes and 22 tRNA genes, whereas the SSC region contains 12 protein coding genes and one tRNA gene. Eleven cp protein coding genes and six tRNA genes contain introns in *F*. *tataricum*. Among the tRNA genes, *trnK*-UUU has the largest intron in both *F*. *tataricum* (2,460 bp) and *F*. *esculentum* (2,458 bp).

**Fig 3:**
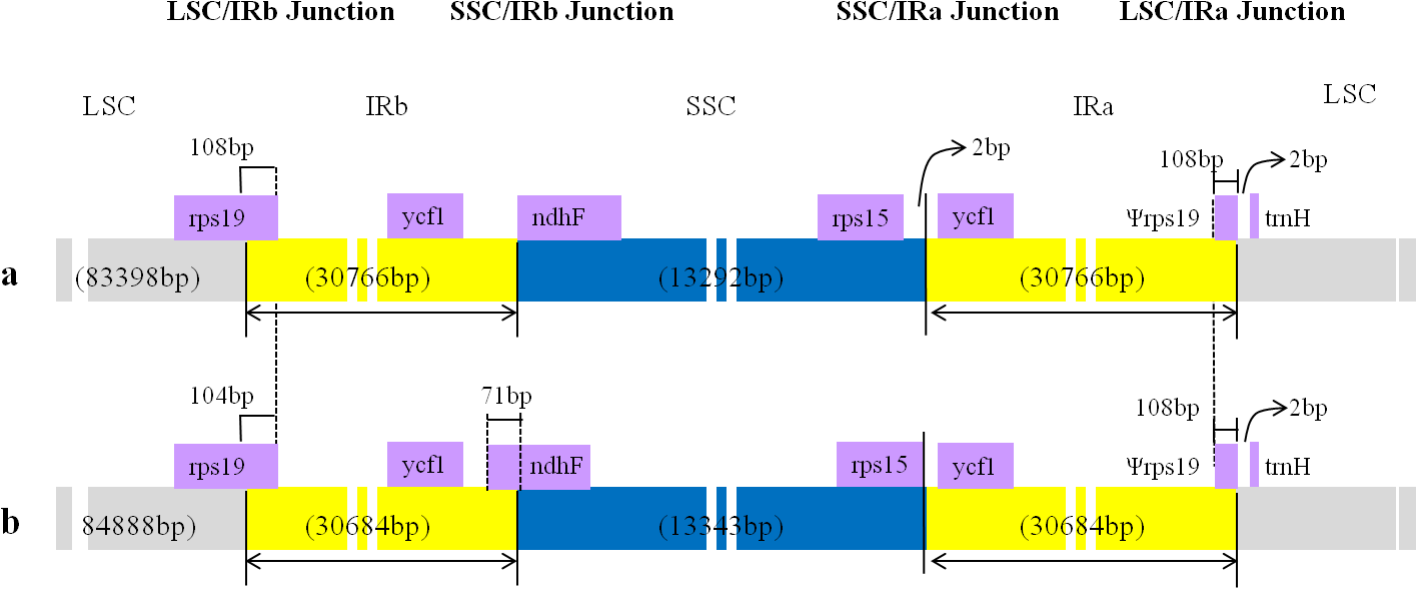
Comparison of the borders of LSC, SSC, and IR regions between the chloroplast genomes of two *Fagopyrum* species. a. *F*. *tataricum* **. b.** *F*. *esculentum*.

The total size variation between *F*. *tataricum* and *F*. *esculentum* cp genomes can be accounted for an 164 bp shorter IR region in *F*. *esculentum*. The border regions of the *F*. *tataricum* and *F*. *esculentum* cp genomes were compared to analyze the expansion variation in junction regions. *Rps19*, *ycf1*, *ndhF*, *rps15*, and *trnH* were found in the junctions of LSC/IR and SSC/IR regions. The *rps19* gene of the LSC in *F*. *tataricum* extended into the IRb region, which created a short pseudo gene of 108 bp at the LSC/IRb junction. This *rps19* pseudo gene is 104 bp in *F*. *esculentum*. The *ndhF* gene of SSC in *F*. *esculentum* extends into the IRb with the initial 71 bp 5′ portion of the gene initiating in the IRb region, whereas this is located at the beginning of the SSC region in *F*. *tataricum*. Similarly, the SSC region of *F*. *esculentum* extended exactly within the *rps15* gene, whereas in *F*. *tataricum* the SSC region extended to 2 bp beyond the *rps15* gene. The location of other genes (e.g., *Ψrps19*, *trnH*, and *ycf1* pseudogene) are similar in both cp genomes (Fig 3).

### Divergence hotspot

The complete cp genomes of *F*. *tataricum* and *F*. *esculentum* were compared and plotted using the mVISTA program to elucidate the level of sequence divergence. The comparison shows that the coding regions of both cp genomes are highly conserved compared to non-coding regions. However, the intergenic region showed the greatest sequence divergence between the two cp genomes. More divergence was found in the sequences of the *trnL*-UAA, *ndhF*, *trnM*-CAU *ndhK*, *petN*, *rpoB*, *trnS*-GCU, and *trnR*-UCU regions, compared to others. The nucleotide and amino acid sequences of protein coding genes of *F*. *tataricum* and *F*. *esculentum* are highly similar with an average sequence similarity of 98.8 and 98.3% respectively. Between the two species, the nucleotide sequence identity of the LSC, SSC, and IR are 96, 99.5, and 99%, respectively. The most conserved genes include the four rRNA genes, along with genes from photosystem I, cytochrome b/f complex, and ATP synthase (S4 Table).

### Divergence of coding gene sequence

**Fig 4:**
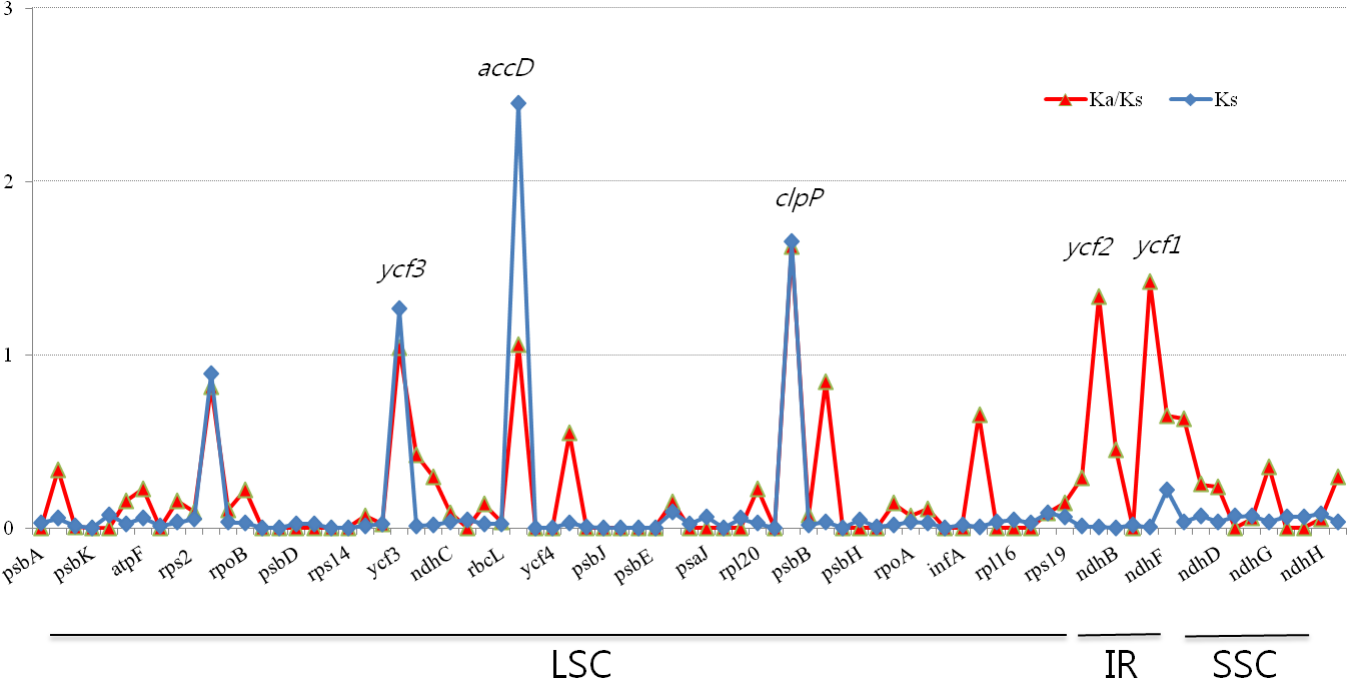
Gene-specific Ks values between the chloroplast genomes of two *Fagopyrum* species (*F*. *tataricum* and *F*. *esculentum*). The Ks value was calculated with PAL2NAL. Four genes (*rpoC2*, *ycf3*, *accD*, and *clpP*) returned Ks values greater than 0.5, whereas the Ks values of the other genes were below 0.5.

The average Ks values between the two buckwheat species are 0.1237, 0.0725, and 0.0088 in the LSC, SSC, and IR regions respectively, with a total average ratio of 0.0683 across all regions (S4 Table). Although the coding region is highly conserved, we observed a slight variation in the divergence of the coding region. Based on the comparison of Ks values among the regions, higher Ks values were observed for some genes, including *rpoC2*, *ycf3*, *accD*, and *clpP*. The Ka to Ks ratio was also calculated, which was >1 for *ycf1* and *ycf2* of IR region and three genes *ycf3*, *accD and clpP* from LSC region (Fig 4).

### Distribution of tandem repeats

**Fig 5:**
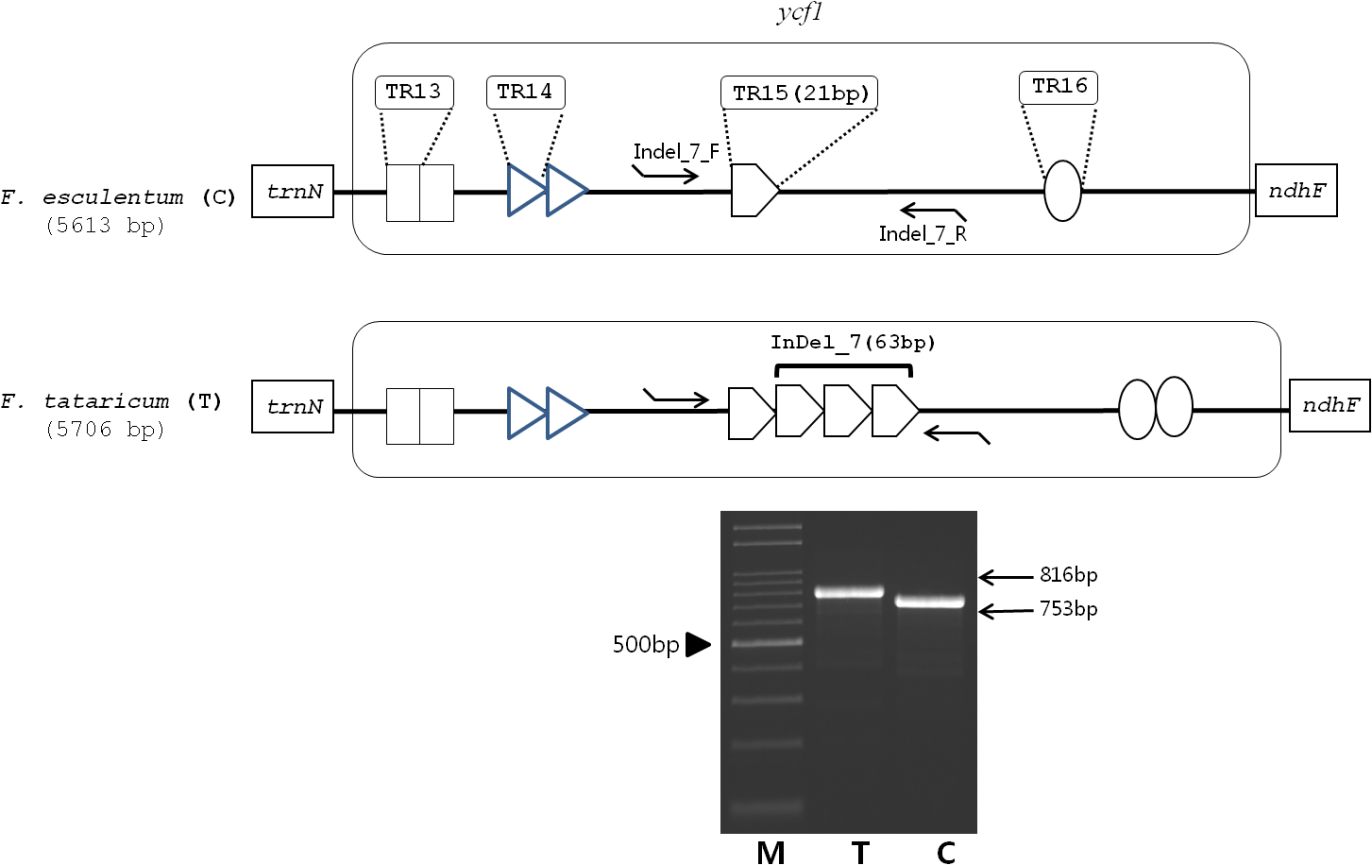
Schematic diagram of the alignment of *F*. *tataricum* and *F*. *esculentum ycf1* genes. Tandem repeats #13, #14, #15 and #16 are designated by a rectangle, triangle, pentagon and circle, respectively. InDel #7 primers that amplify the TR15 region are shown as arrowed lines. M; 100 bp DNA ladder (Bioneer, Daejon, South Korea), T; *F*. *tataricum*, C; *F*. *esculentum*.

Within the cp genome, tandem repeats were compared between *F*. *tataricum* and *F*. *esculentum*. A total of 19 tandem repeats were identified in *F*. *tataricum* and *F*. *esculentum* combined, with varying sizes of repeat units. Of these, 15 were found within intergenic sequences (IGS) and four within coding sequences, all of which shared a similar sequence identity between the two species. These repeating units are repeated from one to four times in both species. Among these repeats, eleven repeats are located in the LSC, seven within in the IR and one in the SSC region (Table 2). Those tandem repeats located in the IR region are highly diverged and the copy number variation of the tandem repeats within the TR15 (Tandem repeat 15 shown in Table 2) in the *ycf1* gene could account for the 63 bp InDel #7 (Fig 5). Similarly, palindromic repeats were also compared between the two species; identifying three and four repeats in *F*. *tataricum* and *F*. *esculentum* respectively. Among the palindormic repeats, four are located in the IGS and one is located in rpl16 intron region of the LSC region. Among these, both species have palindromic repeats at two similar locations, namely the IGS of *rbcL* and *accD* and the IGS of *psbT* and *psbN*. Despite sharing similar locations in the two species, two of the palindromes varied in their loop size (Table 3).

**Table 2:** Comparison of chloroplast genome tandem repeats in *F*. *tataricum* and *F*. *esculentum*.

Tandem Repeat(TR)	Position^a^	Repeat Unit Length(bp)	Repeat Unit Sequences	Repeat numbers of *F*. *esculentum / F*. *tataricum*	Region^b^	Remark
TR1	IGS (*trnI*, *ycf2*)	14	TATTGTATTATACT	2/2	LSC	
TR2	IGS (*rrn4*.*5*, *rrn5*)	32	CATTGTTCAACTCTTTGACAACACCAAAAAAC	2/2	LSC	
TR3	IGS (*psbI*, *trnS*)	13	AAAGATAAATAAA	2/1	LSC	
TR3	IGS (*trnS*, *trnG*)	19	ATAATATAATAATAAACAT	2/2	LSC	
TR4	IGS (*trnY*, *trnE*)	18	ATTTTAAATTTGAGGCGT	2/1	LSC	
TR5	IGS (*ycf3*, *trnS*)	16	TTTATTTTGTTTGTGT	2/1	LSC	
TR6	IGS (*trnT*, *trnL*)	14	AATTAAGTTAAGAT	2/1	LSC	
TR7	IGS (*trnW*, *trnP*)	14	ATATACTATATAGA	2/2	LSC	
TR8	IGS (*clpP*, *psbB*)	15	ATAGAATTCGAATAA	2/2	LSC	
TR9	IGS (*rps4*, *trnT*)	18	TGTCCTAGAACGAATAC	2/2	LSC	
TR10	IGS (*trnW*, *trnP*)	15	TATATACTAAATAGAA	2/2	LSC	
TR11	IGS (*trnI*, *ycf2*)	15	TTGACATTTTCATTG	2/2	LSC	
TR12	IGS (*rps12*,*trnV*)	23	ACCAAACATATGCGGATCCAATC	1/2	IR	
TR13	CDS (*ycf1*)	24	AAATTCGTCTATGATGAACTGCAT	2/2	IR	
TR14	CDS(*ycf1*)	21	ACAAAGTACTTCAATTCTGAC	2/2	IR	
TR15	CDS (*ycf1*)	21	AAACGAAAGAAGAATACTTGC	1/4	IR	Indel 7
TR16	CDS(*ycf1*)	24	TTTGACCTATGGTTTTTTTTTCTT	1/2	IR	
TR17	IGS (*trnV*, *rps12*)	23	GTGATTGGATCCGCATATGTTTG	1/2	IR	
TR18	IGS (*ycf2*, *trnI*)	15	CAATGAAAATATCAA	2/2	IR	
TR19	IGS (*ndhF*, *rpI32*)	13	AGTAACTATTTTC	1/2	SSC	

^a^IGS; Intergenic sequence, CDS; Coding sequence

^b^LSC; Large Single Copy, IR; Inverted Repeat, SSC; Small Single Copy

**Table 3:** Comparison of chloroplast genome palindromic repeats in *F*. *tataricum* and *F*. *esculentum*.

Position^a^	Repeat Unit Length(bp)	Repeat Units Sequences	*F*. *esculentum/F*. *tataricum*		Region^b^
			Repeat numbers	Loop (bp)	
IGS (*petN*, *psbI*)	31	CACTAATCTAATAGATAGTATGGTAGAAAGA	2/0	11/0	LSC
IGS (*rbcL*, *accD*)	23	ATTCGGCTCAATCTTTTTACTAA	2/2	2/1	LSC
IGS (*psbT*, *psbN*)	25	GTTGAAGTAATGAGCCTCCCAATAT	2/2	8/7	LSC
Intron (*rpl16*)	28	TAAGAATTCAAATAAATCTCAAAATATA	2/0	14/0	LSC
IGS (*trnH*, *psbA*)	21	AAATTAAAGGAGCAATACCAA	0/2	0/21	LSC

^a^IGS; Intergenic sequence

^b^ LSC; Large Single Copy

### Authentication of common and bitter buckwheat using InDel markers

**Fig 6:**
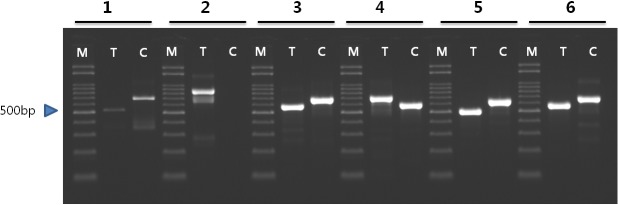
Confirmation of InDel region between the chloroplast genomes of *F*. *esculentum* and *F*. *tataricum* by PCR amplification. M; 100 bp DNA ladder (Bioneer, Daejon, South Korea), T; *F*. *tataricum*, C; *F*. *esculentum*.

**Fig 7:**
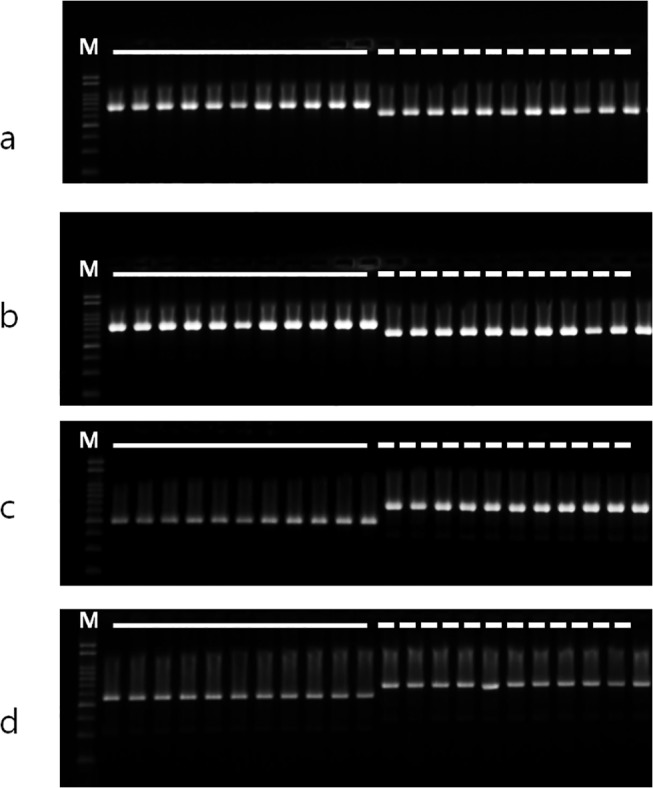
Confirmation of InDel regions between *F*. *esculentum* and *F*. *tataricum* germplasm chloroplast genome by PCR amplification. a, b, c, and d; PCR amplification with InDel #3, InDel #4, InDel #5 and InDel #6 region specific markers (S2 Table), respectively. M; 100 bp DNA ladder (Bioneer, Daejeon, South Korea). Solid line indicates *F*. *tataricum*, dotted line indicates *F*. *esculentum*.

InDel mutations were compared between *F*. *tataricum* and *F*. *esculentum*. A total of seven InDel patterns were identified between the two buckwheat species, most of which were found in IGS regions, with one located in the coding sequence of *ycf1*. The size of the InDels within the IGS ranged from 63 to 175 bp, while that within the *ycf1* coding sequence was 63 bp (S2 Table). The presence of these six InDels in the IGS region was confirmed by PCR amplification in both *F*. *tataricum* and *F*. *esculentum*. PCR analysis of InDel #2 showed a variation in the size of the amplicon in *F*. *esculentum*. InDel #3 to InDel #6 showed identical amplicon sizes and sequences to those of the complete cp sequences (Fig 6). We utilized the PCR markers for the InDel regions #3 to #6 to evaluate the genotypes among large collections of both species: 11 *F*. *tataricum* and 11 *F*. *esculentum* accessions. All tested accessions in each species were identical and concurrently showed clear InDel differences between both species, indicating that these markers could be reliably used for authentication of each of the species (Fig 7).

## Discussion

### Highly efficient NGS technology for chloroplast genome sequencing

Chloroplast DNA sequences are widely used in genetic engineering [24] and in reconstructing evolutionary relationships among plants [11, 25, 26]. Complete cp genome sequences harbor enough information to reconstruct both recent and ancient diversifications. Although some cp genome sequences are available from more than 500 species, the complete cp genome sequence for many important plant species is not available yet [27, 28]. This is due to complete sequencing having been a costly and time-intensive effort, thus limiting sequencing to a small portion of the cp genome, which in many cases is insufficient for determining evolutionary relationships, especially within species or between closely related species. In recent years, new DNA sequencing technologies, termed next-generation sequencers, have made it considerably cheaper and easier to sequence complete cp genomes. While current methods using next-generation sequencers allow up to 48 cp genomes to be sequenced at one time, newer methods will allow potentially hundreds of flowering plant cp genomes to be sequenced at once, significantly reducing the per-sample cost of cp genome sequencing [8]. The powerful and flexible nature of NGS has permeated many areas of study, enabling the development of a broad range of applications that have transformed study designs capable of unlocking information of the genome, transcriptome, and epigenome of any organism. Here, we have obtained the complete and error-free cp genome sequence using low coverage whole genome sequences for *F*. *tataricum* and applied this to a comparative analysis with the cp sequence of the closely related *F*. *esculentum* species.

### Evolution of *F*. *tataricum* and *F*. *esculentum*

Variation in the divergence of the coding region was observed between tartary and common buckwheat species. Although the coding region exhibited a highly conserved nature, *rpoC2*, *ycf3*, *accD*, and *clpP* genes of the LSC region of tartary buckwheat showed a higher evolution rate compared to other genes. Yamane et al. [26] found that the *accD* gene had a high evolution rate in *Fagopyrum* and proposed that this gene was under a weak selection constraint. This is consistent with the high Ks value (2.4538) obtained for *accD* in this study (S4 Table). Other genes we identified as having an unexpectedly high evolution rate between the two studied *Fagopyrum* species include *rpoC2*, *ycf3*, and *clpP*. Cuenoud et al. [25] reported that the *matK* gene has a higher Ks value than *accD*, but in this study, we found the value of matK gene is lower than that of *accD* gene in our investigated samples. Distribution of Ks values indicate that the LSC region is under greater selection pressure than the rest of the cp genome and our data confirm a positive selection pressure and neutral evolution of the protein coding genes. The ratio of non-synonymous (Ka) to synonymous (Ks) value, Ka/Ks ratio, showed a similar pattern to Ks values for most of genes except several genes such as *ycf1* and *ycf2* genes. The *accD*, *clpP*, *ycf3* genes in LSC and *ycf1*, *ycf2* in IR region presented a higher Ka/Ks ratio value (>1.0). The *ycf1* and *ycf2* genes with unclear functions in IR region showed a biased high value for Ka/Ks ratio value compared to Ks value that indicate these two genes evolved at a faster rate in addition to other three genes of LSC. Based on the sequence similarity among the three regions, the IR region is more conserved than the LSC and SSC regions. This is in agreement with earlier reports that hypothesized that the frequent recombinant events occurring in the IR region result in selective constraints on sequence homogeneity, resulting in the IR region diverging at a slower rate than single copy regions [29, 30, 31].

### Biomarkers to differentiate common and bitter buckwheat

The evolutionary InDel hot spots were compared between *F*. *tataricum* and *F*. *esculentum*. Six InDels within IGS regions are identical among accessions of each species and showed clear polymorphism between the *F*. *tataricum* and *F*. *esculentum* species. Meanwhile, there was variation among accessions of *F*. *esculentum* for InDel #2, indicating there is sequence divergence among the wild germplasm of *F*. *esculentum*, even if there is no variation among the domesticated accessions of *F*. *tataricum*. The *F*. *tataricum* reference sequence is that of the wild ancestor of common buckwheat, *F*. *esculentum* Moench subsp. *ancestrale* [17, 32] and analyses implied that in cultivated common buckwheat an InDel region contributed to sequence variation during domestication and resulting in amplicon size variation. PCR analysis of the InDel #3 region in 75 *F*. *tataricum* and 21 *F*. *esculentum* germplasm lines confirmed the presence of the InDel #3 region, which always showed a similar amplicon size (data not shown). All of the tested accessions have different geographical origins and show diversity [33]. This suggests that, although these accessions are from different locations, which differ in the nuclear genome, they share similar cp genomes. This application of PCR analysis to the InDel region can be effectively used as a biomarker to identify varietal contamination in seeds mixtures [34]. Markers, such as DNA polymorphism or specific protein electrophoretic bands, may be analyzed directly using tissues from individual plants or the endosperm of the seed [35]. PCR amplification of the InDel region shown in this study can be efficiently utilized in the identification procedure of buckwheat seed mixture containing two species (i.e., *F*. *tataricum* and *F*. *esculentum*). It can also be applied for authentication of the raw materials used for high value processed foods, such as buckwheat tea and buckwheat noodles that contain various proportions of the two buckwheat species. Yoon *et al*., [36] used starch granule associated proteins (SGAPs) as a biomarker to identify the botanical origin of starches used in noodle manufacture. Dietary material prepared from *F*. *tataricum* is more beneficial than those prepared from *F*. *esculentum* because tartary buckwheat is a richer source of rutin compared to common buckwheat, which helps in reducing intra-vascular cholesterol, high blood pressure, and diabetes, and is also reported to have a crucial role in pharmaceutical research [13]. Hence, PCR analysis of the InDel region described here can be utilized as a biomarker to differentiate between *F*. *tataricum* and *F*. *esculentum* in raw materials or processed buckwheat products.

## Conclusion

We present the first report of the complete cp genome sequence of *F*. *tataricum* and describe the evolutional relationship between the two cultivated buckwheat species. We also describe useful InDel markers that could be applied for the authentication of these buckwheat species, which have different food values.

## Supporting Information

S1 Table List of buckwheat germplasm accession numbers used in this study. (XLSX)

S2 Table Primers list for InDel validation between the chloroplast genomes of F. tataricum and *F*. *esculentum*. (XLSX)

S3 Table List of genes in *F*. *tataricum* chloroplast genome. (XLSX)

S4 Table Nucleotide and amino acid sequence, Ks and Ka values of chloroplast genome genes between *F*. *tataricum* and *F*. *esculentum*. (XLSX)
